# A common founder for the V126D *CDKN2A* mutation in seven North American melanoma-prone families

**DOI:** 10.1054/bjoc.2001.1944

**Published:** 2001-08

**Authors:** A M Goldstein, L Liu, M G Shennan, D Hogg, M A Tucker, J P Struewing

**Affiliations:** Division of Cancer Epidemiology and Genetics, National Cancer Institute, Bethesda, Maryland, 20892, USA; Department of Medicine, The University of Toronto, Toronto, Ontario, M5S 1A8, Canada; Department of Division of Medical Oncology, Toronto-Sunnybrook Regional Cancer Centre, Toronto, Ontario, M4N 3M5, Canada; Department of Medical Biophysics, Toronto-Sunnybrook Regional Cancer Centre, Toronto, Ontario, M4N 3M5, Canada

**Keywords:** melanoma, *CDKN2A*, V126D, founder

## Abstract

One of the most common melanoma-related *CDKN2A* mutations reported in North America is the V126D mutation. We examined nine markers surrounding *CDKN2A* in three American and four Canadian families carrying the V126D mutation. All seven families had a haplotype consistent with a common ancestor/founder for this mutation. In addition, the mutation appears to have originated 34–52 generations ago (1-LOD-unit support interval 13–98 generations). © 2001 Cancer Research Campaign http:///www.bjcancer.com


British Journal of Cancer (2001) 85(4), 527–530
© 2001 Cancer Research Campaign
doi: 10.1054/ bjoc.2001.1944, available online at http://www.idealibrary.com on IDEAL

http://www.bjcancer.com

### A common founder for the V126D *CDKN2A* mutation in seven North American melanoma-prone families

**AM Goldstein[1], L Liu[2], MG Shennan[3], D Hogg[2,4], MA Tucker[1] and JP Struewing[1]**

[1]Division of Cancer Epidemiology and Genetics, National Cancer Institute, Bethesda, Maryland 20892, USA; Departments of [2]Medicine and [4]Medical Biophysics, The University of Toronto, Toronto, Ontario M5S 1A8, Canada; [3]Division of Medical Oncology, Toronto-Sunnybrook Regional Cancer Centre, Toronto, Ontario M4N 3M5, Canada

**Summary** One of the most common melanoma-related *CDKN2A* mutations reported in North America is the V126D mutation. We examined nine markers surrounding *CDKN2A* in three American and four Canadian families carrying the V126D mutation. All seven families had a haplotype consistent with a common ancestor/founder for this mutation. In addition, the mutation appears to have originated 34–52 generations ago (1-LOD-unit support interval 13–98 generations). © 2001 Cancer Research Campaign http:///www.bjcancer.com

**Keywords**: melanoma, *CDKN2A*, V126D, founder

Cutaneous malignant melanoma (CMM) is a potentially fatal form of skin cancer with a complex aetiology (Chin et al, 1998). Approximately 10% of melanomas arise in individuals with a familial predisposition to the disease, and a subset of these kindreds has an inherited susceptibility for their melanoma. The most common genetic lesion comprises a germline mutation in the *CDKN2A* gene, which encodes two unrelated cell cycle regulatory proteins: $p16^{INK4A}$ and $p14^{ARF}$ (Serrano et al, 1993; 1995; Zhang et al, 1998; Pomerantz et al, 1998). Mutations that affect the function of $p16^{INK4A}$ occur in approximately 20% of melanoma-prone families worldwide; the mutation detection frequency rises to ≥ 50% in kindreds with more than 6 CMM patients (Kefford et al, 1999; Goldstein and Tucker, 2001).

Many different germline *CDKN2A* mutations have been identified in melanoma-prone families from North America, Europe, and Australasia. The majority of mutations so far identified are missense mutations scattered throughout the *CDKN2A* coding region. Although some mutations have been observed only once, numerous mutations have repeatedly been found in different families. Haplotype analyses of common recurrent mutations from the same geographic areas (e.g. 225del19 from the Netherlands, 113insArg from Sweden, G-34T from Canada) (Gruis et al, 1995; Borg et al, 1996; Liu et al, 1999) or geographically diverse areas (e.g. M531, 23ins24, G101W) (Pollock et al, 1998; Liu et al, 1999; Ciotti et al, 2000) revealed that the vast majority of these recurrent mutations result from a single genetic origin, i.e. the mutations derive from common founders or ancestors.

One of the most common *CDKN2A* mutations reported in North America is the V126D mutation. This mutation inhibits the catalytic activity of the cyclin D1/CDK4 and cyclin D1/CDK6 complexes in vitro (Ranade et al, 1995). It was also shown to be temperature sensitive for binding to CDK4 and CDK6 in vitro, for inhibiting cyclin D1-CDK4 in a reconstituted pRb-kinase assay, and for increasing the proportion of G1-phase cells following transfection (Parry and Peters, 1996). Previous examination of three American melanoma-prone families with this mutation suggested the possibility of a common haplotype (Goldstein et al, 2000). We have now examined additional markers in these three families, including marker D9S974, a marker extremely close to the *CDKN2A* gene (Randerson-Moor et al, 2001). In addition, we have examined nine markers surrounding the *CDKN2A* gene in four Canadian families carrying the V126D mutation. The results show that all seven families have a haplotype consistent with a common founder for this mutation. In addition, although based on only seven families, the mutation appears to have originated approximately 34–52 generations ago (1-LOD-unit support interval 13–98 generations).

### SUBJECTS AND METHODS

### Families

Details about the seven North American families have been presented previously (Hussussian et al, 1994; Goldstein et al, 2000; Liu et al, 1999). For all participants, written informed consent was obtained prior to participation under Institution Review Board approved protocols. Briefly, the four Canadian families had an average of three melanoma patients per family (Table 1). In addition, patients from two of the families had multiple primary melanomas. The three American families all had patients with multiple primary melanomas; two of the families had at least one patient with pancreatic cancer. Although six of the seven families had German/English ancestries, little is known about the ancestral pathway for melanoma in these families prior to their arrival in North America.

### Genotyping

Nine markers were genotyped for the analysis to determine which alleles from loci flanking *CDKN2A* were transmitted with CMM

*Received 16 February 2001*
*Revised 22 May 2001*
*Accepted 30 May 2001*

*Correspondence to:* AM Goldstein

**Table 1** North American families with V126D *CDKN2A* germline mutation. Family ancestry, number of CMM patients, presence of multiple primary melanoma tumours, presence of pancreatic cancer, number of tested V126D mutation carriers, and number of family members genotyped for haplotype analysis. American families: K, J, L; Canadian families: 101, 102, 103, 104

| Families | American | | | Canadian | | | |
|---|---|---|---|---|---|---|---|
| | K | J | L | 101 | 102 | 103 | 104 |
| Family ancestry | English/German/ Scandinavian | German/ English | German/Dutch/ English | German/ English | Unknown | German/ English | German/English/ French |
| No. of members w/CMM | 5 | 6 | 10 | 3 | 2 | 4 | 3 |
| Multiple primaries (Y/N) | Y | Y | Y | N | Y | N | Y |
| Pancreatic cancer (Y/N) | Y ($n$ = 3) | N | Y ($n$ = 1) | N | N | N | N |
| No. of tested V126D carriers | 6 | 7 | 5 | 2 | 1 | 1 | 2 |
| No. of genotyped subjects:* | 6 | 5 | 3 | 3 | 1 | 1 | 2 |
| No. affected | 3 | 3 | 3 | 2 | 1 | 1 | 2 |
| No. unaffected | 3 | 2 | 0 | 1 | 0 | 0 | 0 |

*Used in haplotype analysis.

in each of the families: IFNA, D9S736, D9S1749, D9S974, D9S942, D9S1748, D9S1604, D9S171, and D9S126. Markers D9S1749 (approximately 0.0105 M or 1.05 cM distal) and D9S974 (approximately 0.012 cM proximal) were the closest flanking markers to exon 2 of the *CDKN2A* gene (Randerson-Moor et al, 2001). Allele sizes for all markers are comparable with those from haplotype studies of Ciotti et al (2000) and Pollock et al (except for D9S736 and D9S126) (1998).

### Dating the mutation

To estimate when the V126D mutation originated, we used a maximum likelihood (MLE) method developed by D. Goldgar (Neuhausen et al, 1996, 1998) and previously applied to G101W (Ciotti et al, 2000). Briefly, the joint likelihood of the V126D haplotypes was written as a function of the recombination fraction between the disease and each marker, the number of generations (G) since the mutation arose, and the mutation rate (0.0006 for all markers except D9S1749 [0.01] and D9S942 [0.002]) and allele frequencies at each marker locus (Ciotti et al, 2000) (Table 2). The MLE method was used to find the value of G that best fitted the pattern of haplotype sharing at the nine marker loci. When haplotypes could not be determined with certainty, all possible haplotypes consistent with the observed multilocus genotypes were considered in the analysis. Approximate support intervals were calculated by finding the value of G on either side of the most likely value that had a ≥ 10-fold decrease in likelihood.

### RESULTS

Haplotype analysis using 9 polymorphic markers spanning the *CDKN2A* locus was performed on index cases and additional family members (when available) to determine whether carriers from the seven families harbored the same mutation identically by descent. Table 3 shows the disease haplotypes or genotypes for the seven families. Both alleles are indicated for markers for which segregating alleles could not be unambiguously determined. All seven families showed a haplotype or genotype consistent with a single genetic origin for the V126D mutation. The D9S1749-D9S1604 haplotype 16/17–6–11–9–2 appears to be common across all families, after allowing for recombination over time. One American family (K) had the 5 allele at D9S974, rather than the 6 allele seen in all other families. D9S1749, previously shown to vary in allele size because of replication slippage resulting in the loss or gain of one or more repeat units during meiosis (Pollock et al, 1998), showed either the 16 or 17 allele co-segregating in all but one family. Family 103 showed allele 14 co-segregating with CMM. Allowing for replication slippage in D9S1749, families J and L shared a common haplotype from IFNA to D9S126. It was not possible to further assess the extended sharing of the disease related haplotype in other families because the co-segregating alleles could not be unequivocally determined.

Results from the MLE method suggested that the V126D mutation originated approximately 34–52 generations ago (1-LOD-unit support interval 13–98 generations) or approximately 680–1040 years ago (1-LOD-unit support interval 260–1960 years) using a 20-year generation interval or 1020 to 1560 years ago (1-LOD-unit support interval 390–2940 years) using a 30-year generation interval. The maximum likelihood estimates for alleles 16 and 17 of D9S1749 were equivalent; thus a range in the estimate of the mutation origin is presented (e.g. 34–52 generations).

### DISCUSSION

The V126D mutation appears to have originated from a common founder or ancestor, as is the case with most recurrent *CDKN2A* mutations studied to date. Only 23ins24, a 24 base-pair duplication, has been shown to have multiple origins (Pollock et al, 1998), probably due to the inherent instability of the wild type *CDKN2A* 5’ tandem repeat region. Although there are many recurrent *CDKN2A* mutations, only two – G101W and 113insArg – have been evaluated to determine their ages of origination. Using the same MLE methods as was used in the current study, Ciotti et al (2000) and Hashemi et al (2001) concluded that the G101W and 113insArg mutations both originated approximately 100 generations ago. Using the same maximum likelihood estimate method, we estimated that the V126D mutation originated approximately 34–52 generations ago (1-LOD-unit support interval 13–98 generations). Given the relatively small number of families in the present study and the sensitivity of the MLE method to the marker mutation rates, we also employed an approach proposed by Neuhausen et al (1996) to evaluate the variability in the estimated age of the mutation. The age of origin for the mutation was re-estimated assuming marker mutation rates that were an order of magnitude (i.e. 10x) lower and higher (Neuhausen et al, 1996) than the values used in the original analysis. The estimated age of the V126D mutation was reduced to 9 generations when a 10-fold increase in marker mutation rates was assumed and 71 generations

 © 2001 Cancer Research Campaign

**Table 2** Allele frequencies at each marker locus (from Ciotti et al, 2000) for dating the mutation

| Allele No. | IFNA | D9S736 | D9S1749 | D9S974 | D9S942 | D9S1748 | D9S1604 | D9S171 | D9S126 |
|---|---|---|---|---|---|---|---|---|---|
| 1 | 0.08 | 0.06 | 0.000 | 0.064 | 0.012 | 0.00 | 0.44 | 0.280 | 0.01 |
| 2 | 0.15 | 0.09 | 0.000 | 0.038 | 0.050 | 0.00 | 0.56 | 0.073 | 0.11 |
| 3 | 0.30 | 0.09 | 0.000 | 0.038 | 0.025 | 0.08 | | 0.050 | 0.11 |
| 4 | 0.11 | 0.42 | 0.000 | 0.090 | 0.025 | 0.16 | | 0.061 | 0.21 |
| 5 | 0.08 | 0.19 | 0.000 | 0.103 | 0.138 | 0.11 | | 0.305 | 0.17 |
| 6 | 0.22 | 0.15 | 0.000 | 0.180 | 0.100 | 0.06 | | 0.012 | 0.29 |
| 7 | 0.06 | | 0.012 | 0.220 | 0.025 | 0.15 | | 0.085 | 0.10 |
| 8 | | | 0.001 | 0.090 | 0.160 | 0.21 | | 0.050 | |
| 9 | | | 0.001 | 0.100 | 0.088 | 0.12 | | 0.024 | |
| 10 | | | 0.001 | 0.013 | 0.038 | 0.10 | | 0.060 | |
| 11 | | | 0.050 | 0.064 | 0.075 | 0.01 | | | |
| 12 | | | 0.025 | | 0.012 | | | | |
| 13 | | | 0.025 | | 0.038 | | | | |
| 14 | | | 0.075 | | 0.001 | | | | |
| 15 | | | 0.087 | | 0.000 | | | | |
| 16 | | | 0.062 | | 0.012 | | | | |
| 17 | | | 0.062 | | 0.125 | | | | |
| 18 | | | 0.075 | | 0.025 | | | | |
| 19 | | | 0.087 | | 0.038 | | | | |
| 20 | | | 0.012 | | 0.000 | | | | |
| 21 | | | 0.025 | | 0.000 | | | | |
| 22 | | | 0.062 | | 0.000 | | | | |
| 23 | | | 0.075 | | 0.013 | | | | |
| 24 | | | 0.038 | | | | | | |
| 25 | | | 0.050 | | | | | | |
| 26 | | | 0.025 | | | | | | |
| 27 | | | 0.050 | | | | | | |
| 28 | | | 0.025 | | | | | | |
| 29 | | | 0.001 | | | | | | |
| 30 | | | 0.012 | | | | | | |
| 31 | | | 0.012 | | | | | | |
| 32 | | | 0.050 | | | | | | |

**Table 3** North American families with V126D *CDKN2A* germline mutation. Haplotype analysis for 9p markers

| Familiest | Haplotype/genotype for each family | | | | | | |
|---|---|---|---|---|---|---|---|
| | American | | | Canadian | | | |
| | K | J | L | 101 | 102 | 103 | 104 |
| Markers | | | | | | | |
| IFNA | 6 | 6 | 6 | 6 | 3,6 | 3,6 | 3 |
| D9S736 | 4 | 1 | 1 | 1,4 | 1,5 | 4 | 4,5 |
| D9S1749 | **17**,18* | **16**** | **17** | **16** | **16**,25 | 14 | **16** *** |
| D9S974 | 5 | **6** | **6** | **6** | **6** | 2,**6** | **6**,7 |
| D9S942 | **11** | **11** | **11** | **11** | 8,**11** | **11**,19 | **11**,16 |
| D9S1748 | **9** | **9** | **9** | **9** | 7,**9** | 8,**9** | 5, **9** |
| D9S1604 | **2** | **2** | **2** | **2** | 1,**2** | 1,**2** | **2** |
| D9S171 | 5 | 1 | 1,5 | 1 | 1,9 | 1 | 1 |
| D9S126 | 4,6 | 4 | 4 | 6 | 4,6 | 4,6 | 4,6 |

*Both alleles are indicated for markers for which segregating alleles could not be unequivocally determined.
**Alleles that are part of the common disease-related haplotype are shown in boldface.
***Line between D9S1749 and D9S974 represents location of V126D mutation.

when a 10-fold decrease in rates was applied (combined 1-LOD-unit support interval 3–145 generations). These additional findings in conjunction with the original results suggest the possibility of a more recent origin for V126D relative to that seen for the G101W and 113insArg mutations. We cannot, however, preclude a more remote origin based on the greater imprecision in the estimated age resulting from the smaller numbers of families available for the current study.

American family K had the 5 allele at D9S974, rather than the 6 allele seen in all the other families. Since D9S974 is the marker closest to the V126D mutation (0.012 cM), a recombination event so close to the mutation would likely indicate a remote origin for the mutation. Replication slippage, conversely, does not necessarily imply an ancient origin for the mutation. Unfortunately it is not possible to determine whether the 5 allele, only 2 base pairs smaller than the consensus 6 allele, resulted from recombination or

*© 2001 Cancer Research Campaign*

replication slippage. Thus, this allele change provides little additional evidence for helping determine the origin of the V126D mutation.

Most recurrent *CDKN2A* mutations observed in North America can be traced back to a European country or region of origin. For example, the recurrent M53I mutation, which has been found at high frequency in North America, Great Britain, and Australia, appears to have originated in Great Britain (Pollock et al, 1998; Liu et al, 1999). Similarly, the G101W mutation, which is very common in the United States, appears to have originated in southwestern Europe; it is the most common mutation detected in France and Italy (Ciotti et al, 2000; Soufir et al, 1998; Ghiorzo et al, 1999; Ruiz et al, 1999). In contrast, the V126D mutation does not appear at high frequency in any other countries besides the United States and Canada. The mutation has been observed in Australia, France and Italy but only rarely in each of these countries. This phenomenon may reflect selective mutation testing in the various countries or it may be related to the origination of this particular mutation. For example, the major *CDKN2A* mutation testing from Great Britain to date has occurred in Northcentral England and Scotland where the V126D mutation has not been observed (MacKie et al, 1998; Newton Bishop et al, 1999). Also, very little data from Germany on *CDKN2A* mutation testing has been published. Testing in other areas of Great Britain, Germany or other parts of continental Europe, however, might reveal the mutation. Although six of the seven families immigrated to North America from Germany and England, the ancestral pathway for melanoma in these families cannot be determined. Additional families from North America as well as from other geographic areas may help determine the geographic origin for this recurrent yet puzzling *CDKN2A* mutation.

### ACKNOWLEDGEMENTS

We wish to thank the participating families, whose generosity and cooperation have made this study possible. We thank Mary Fraser, Laura Fontaine, and Deborah Zametkin for nursing support. We acknowledge the Biomedical Supercomputing Center of the NCI-Frederick Cancer Research and Development Center for allocation of computing time and staff support.

### REFERENCES

Borg A, Johansson U, Johansson O, Hakansson S, Westerdahl J, Masback A, Olsson H and Ingvar C (1996) Novel germline p16 mutation in familial melanoma in southern Sweden. *Cancer Res* **56**: 2497–2500

Chin L, Merlino G and DePinho RA (1998) Malignant melanoma: modern black plague and genetic black box. *Genes Dev* **12**: 3467–3481

Ciotti P, Struewing JP, Mantelli M, Chompret A, Avril M-F, Santi PL, Tucker MA, Bianchi-Scarra G, Bressac-de Paillerets B and Goldstein AM (2000) A single genetic origin for the G101W CDKN2A mutation in 20 melanoma-prone families. *Am J Hum Genet* **67**: 311–319

Ghiorzo P, Ciotti P, Mantelli M, Heouaine A, Queirolo P, Rainero ML, Ferrari C, Santi PL, De Marchi R, Farris A, Ajmar F, Bruzzi P and Bianchi-Scarra G (1999) Characterization of ligurian melanoma families and risk of occurrence of other neoplasia. *Int J Cancer* **12**: 441–448

Goldstein AM, Struewing JP, Chidambaram A, Fraser MC and Tucker MA (2000) Genotype-phenotype relationships in American melanoma-prone families with CDKN2A and CDK4 mutations. *J Natl Cancer Inst* **92**: 1006–1010

Goldstein AM and Tucker MA (2001) Familial melanoma and its management. In: *Genetic Predisposition to Cancer*, 2nd Edition, Eeles R, Easton D, Eng C and Ponder B (eds) (in press). Arnold: London

Gruis NA, van der Velden PA, Sandkuijl LA, Prins DE, Weaver-Feldhaus J, Kamb A, Bergman W and Frants RR (1995) Homozygotes for CDKN2A (p16) germline mutation in Dutch familial melanoma kindreds. *Nature Genet* **10**: 351–353

Hashemi J, Bendahl P-O, Sandberg T, Platz A, Linder S, Stierner U, Olsson H, Ingvar C, Hansson J and Borg Å (2001) Haplotype analysis and age estimation of the 113 insArg CDKN2A founder mutation in Swedish melanoma families. *Genes Chromosomes Cancer* (in press)

Hussussian CJ, Struewing JP, Goldstein AM, Higgins PAT, Ally DS, Sheahan MD, Clark Jr WH, Tucker MA and Dracopoli NC (1994) Germline p16 mutations in familial melanoma. *Nature Genet* **8**: 15–21

Kefford RF, Newton Bishop JA, Bergman W and Tucker MA (1999) Counseling and DNA testing for individuals perceived to be genetically predisposed to melanoma: a consensus statement of the Melanoma Genetics Consortium. *J Clin Oncol* **17**: 3245–3251

Liu L, Dilworth D, Gao L, Monzon J, Summers A, Lassam N and Hogg D (1999) Mutation of the CDKN2A 5′ UTR creates an aberrant initiation codon and predisposes to melanoma. *Nature Genet* **21**: 128–132

MacKie RM, Andrew N, Lanyon WG and Connor JM (1998) CDKN2A germline mutations in U.K. patients with familial melanoma and multiple primary melanomas. *J Invest Dermatol* **111**: 269–272

Neuhausen SL, Mazoyer S, Friedman L, Stratton M, Offit K, Caligo A, Tomlinson G, Cannon-Albright L, Bishop T, Kelsell D, Solomon E, Weber B, Couch F, Struewing J, Tonin P, Durocher F, Narod S, Skolnick MH, Lenoir G, Serova O, Ponder B, Stoppa-Lyonnet D, Easton D, King M-C and Goldgar DE (1996) Haplotype and phenotype analysis of six recurrent BRCA1 mutations in 61 families: results of an international study. *Am J Hum Genet* **58**: 271–280

Neuhausen SL, Godwin AK, Gershoni-Baruch R, Schubert E, Garber J, Stoppa-Lyonnet D, Olah E, Csokay B, Serova O, Lalloo F, Osorio A, Stratton M, Offit K, Boyd J, Caligo MA, Scott RJ, Schofield A, Teugels E, Schwab M, Cannon-Albright L, Bishop T, Easton D, Benitez J, King M-C, Ponder BAJ, Weber B, Devilee P, Borg A, Narod SA and Goldgar D (1998) Haplotype and phenotype analysis of nine recurrent BRCA2 mutations in 111 families: results of an international study. *Am J Hum Genet* **62**: 1381–1388

Newton Bishop JA, Harland M, Bennett DC, Bataille V, Goldstein AM, Tucker MA, Ponder BAJ, Cuzick J, Selby P and Bishop DT (1999) Mutation testing in melanoma families: INK4A, CDK4, and INK4D. *Br J Cancer* **80**: 295–300

Parry D and Peters G (1996) Temperature-sensitive mutants of p16CDKN2 associated with familial melanoma. *Mol Cell Biol* **16**: 3844–3852

Pollock PM, Spurr N, Bishop T, Newton-Bishop J, Gruis N, van der Velden PA, Goldstein AM, Tucker MA, Foulkes WD, Barnhill R, Haber D, Fountain J and Hayward NK (1998) Haplotype Analysis of two recurrent CDKN2A mutations in 10 melanoma families: evidence for common founders and independent mutations. *Hum Mut* **11**: 424–431

Pomerantz J, Schreiber-Agus N, Liegeois NJ, Silverman A, Alland L, Chin L, Potes J, Chen K, Orlow I, Lee HW, Cordon-Cardo C and DePinho RA (1998) The Ink4a tumor suppressor gene product, p19Arf, interacts with MDM2 and neutralizes MDM2's inhibition of p53. *Cell* **92**: 713–723

Ranade K, Hussussian CJ, Sikorski RS, Varmus HE, Goldstein AM, Tucker MA, Serrano M, Hannon GJ, Beach D and Dracopoli NC (1995) Mutations associated with familial melanoma impair p16INK4 function. *Nature Genet* **10**: 114–116

Randerson-Moor JA, Harland M, Williams S, Cuthbert-Heavens D, Sheridan E, Aveyard J, Sibley K, Whitaker L, Knowles M, Newton Bishop J and Bishop DT (2001) A germline deletion of $p14^{ARF}$ but not *CDKN2A* in a melanoma-neural system tumour syndrome family. *Hum Molec Genet* **10**: 55–62

Ruiz A, Puig S, Malvehy J, Lazaro C, Lynch M, Gimenez-Arnau AM, Puig L, Sanchez-Conejo J, Estivill X and Castel T (1999) CDKN2A mutations in Spanish cutaneous malignant melanoma families and patients with multiple melanoma and other neoplasia. *J Med Genet* **36**: 490–493

Serrano M, Hannon GJ and Beach D (1993) A new regulatory motif in cell cycle control causing specific inhibition of cyclin D/CDK4. *Nature* **366**: 704–707

Serrano M, Gomez-Lahoz E, DePinho RA, Beach D and Bar-Sagi D (1995) Inhibition of ras-induced proliferation and cellular transformation by p16INK4. *Science* **267**: 249–252

Soufir N, Avril MF, Chompret A, Demenais F, Bombled J, Spatz A, Stoppa-Lyonnet D, the French Familial Melanoma Study Group, Benard J and Bressac-de Paillerets B (1998) Prevalence of p16 and CDK4 germline mutations in 48 melanoma-prone families in France. *Hum Mol Genet* **7**: 209–216

Zhang Y, Xiong Y and Yarbrough WG (1998) ARF promotes MDM2 degradation and stabilizes p53: ARF-INK4a locus deletion impairs both the Rb and p53 tumor suppression pathways. *Cell* **92**: 725–734

 © 2001 Cancer Research Campaign
